# Vigilance Data in Organ Donation and Solid Organ Transplantation in Germany: Six Years of Experience 2016–2022

**DOI:** 10.3389/ti.2023.11610

**Published:** 2023-09-04

**Authors:** Klaus Böhler, Axel Rahmel, Ana Paula Barreiros

**Affiliations:** Deutsche Stiftung Organtransplantation, Frankfurt am Main, Germany

**Keywords:** organ donation, organ transplantation, disease transmission, donor-transmitted cancer, serious adverse event, serious adverse reaction

## Abstract

The reporting of serious adverse events (SAE) and serious adverse reactions (SAR) is an essential part of an effective vigilance and surveillance system (V&S) in organ donation and transplantation. All SAE and SAR reported to the German organ procurement organization (DSO) between 2016 and 2022 were analyzed. In case of a possible transmission of a disease to one or more recipients, an assessment of imputability was done according to the grading system of the US Disease Transmission Advisory Committee (DTAC). 543 SAE and SAR cases were reported to the DSO and analyzed in detail. 53 of the 543 reports (9.8%) were proven or probable (P/P) transmissions of infectious diseases, malignancies or other diseases to 75 recipients. Infections were the most frequently reported P/P disease transmission occurrences (30/53, 57%). In case of disease transmission, the mortality of the recipients was high (17/75, 23%), especially when a malignant disease was transmitted (11/22, 50 %). Donor-Derived disease transmission is a rare event (53/8,519; 0.6 %), but when it occurs can lead to significant morbidity and mortality.

## Graphical Abstract

**Figure:**
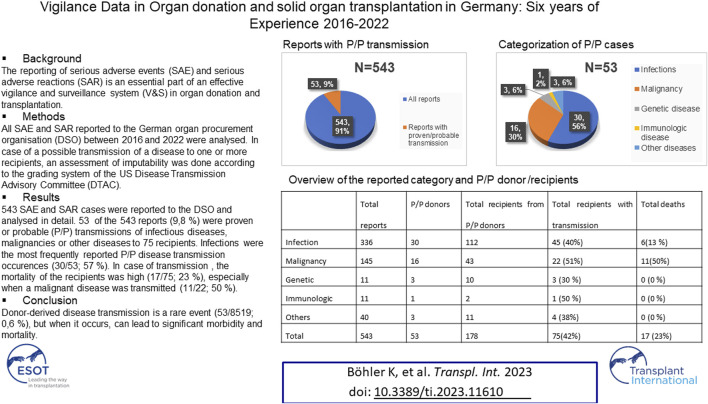

## Introduction

Due to the shortage of organs for solid organ transplantation, different strategies have been developed to increase the donor pool, including the use of organs from expanded-criteria donors and from increased risk donors [1–3]. Compared to the high number of transplantations performed worldwide each year, the number of reported adverse outcomes seems low. Nevertheless donor-derived transmission of infectious diseases and malignancies pose an additional risk to the organ recipients with significant morbidity and mortality [4–9].

As organ donation and transplantation is a complex process involving many different institutions at various steps of the process, an effective vigilance and surveillance system (V&S) is of utmost importance for reducing risks to the recipients [10,11].

The EU-directive 2010/53/EU of 7 July 2010, on standards of quality and safety of human organs intended for transplantation, requires that the member states establish a rapid alert system to report, investigate, register, and transmit relevant and necessary information concerning serious adverse events (SAEs) and serious adverse reactions (SARs) to the involved transplantation centers and the national competent authorities [12].

In this context, the EU-directive defines a serious adverse event as “any undesired and unexpected occurrence associated with any stage of the chain from donation to transplantation that might lead to the transmission of a communicable disease, to death or life- threatening, disabling or incapacitating conditions for patients or which results in, or prolongs, hospitalization or morbidity” [12]. A serious adverse reaction is defined as “an unintended response, including a communicable disease, in the living donor or in the recipient that might be associated with any stage of the chain from donation to transplantation that is fatal, life-threatening, disabling, incapacitating, or which results in, or prolongs, hospitalization or morbidity” [12].

In short, an SAE refers to a serious risk of harm to the recipient although no harm has occurred or been identified yet, whereas an SAR refers to serious harm that has already occurred to one or more recipients and might be associated with the donor.

Setting up a V&S system must be distinguished from a quality management system in organ donation and transplantation. A quality management system sets up a whole range of predefined quality indicators such as occurrence of primary graft failure or in-hospital mortality post-transplant. In a V&S system there are no predefined indicators, instead there are different events and reactions in the whole chain of the process that are not previously known and may influence the quality and safety of organ donation and transplantation. The clear definitions of SAE and SAR described above must be complied with and well understood.

The different steps of a V&S system consist of alerting, reporting, assessing, and managing SAEs and SARs, followed by the surveillance of the recipients. To fulfill the different tasks, procedural rules are determined by the directive 2012/25/EU of 9 October 2012 [13].

According to this directive the member states have to appoint qualified and trained staff for the assessment and processing of the incoming reports, available 24 h/7 days/365 days. The dedicated staff has to alert without any delay the involved transplantation centers, organ procurement organizations, and in case of cross-border organ exchange, the national authorities. An initial report has to be sent to the above-mentioned institutions in order to set up preventive and/or therapeutic measures for the involved recipients. Furthermore, when additional information becomes available following the initial report, it shall be transmitted to the involved institutions. Within 3 months, a final report including the result of the assessment and investigation, as well as the actions taken, should be provided to the relevant parties. If applicable in the individual case, preventive and corrective measures to avoid similar incidents in the future should also be included in the report [13].

In Germany, the organ procurement organization (Deutsche Stiftung Organtransplantation—DSO) is the delegated body assigned by the German competent authority (Federal Ministry of Health) responsible for managing and performing the V&S system in organ donation and transplantation.

On a national level, the regulation of the law of donation, removal, and transplantation of organs and tissues of 28th May 2014 implemented the V&S system according to both directives [14].

Analyses of reported SAEs and SARs should help with identifying risks in the process of donation and transplantation. Ideally, the risk analyses will be integrated into adaptations of new guidelines or standard operating procedures (SOP). Sharing the information of known SAE and SAR cases between all involved parties like donor hospitals, transplantation centers, procurement organizations, organ exchange organization, and national health authorities is crucial. All these efforts are important to enable maximal recipient safety and security.

## Materials and Methods

All incoming reports of SAEs and SARs were assessed by a special team of qualified and trained physicians of the DSO (SAE/SAR team of the DSO). The team consists of one executive physician, one staff physician, and nine physicians from the seven different German regional sections who also worked as organ procurement coordinators but with a focus on SAE and SAR. Furthermore, there was support from designated external experts in the field (e.g., virology, hematology, pathology) provided in a case-by-case decision to help review the cases. All procedural steps were carried out in accordance with the directive 2012/25/EU [13].

As a first step, the reports were grouped into five categories: pathogens/infections, suspected malignancies, genetic diseases, immunologic events/reactions, and other diseases.

All reports were classified as SAE or SAR according to the definitions of the directive [12]. For every reported SAR, an assessment of imputability was carried out, grading the probability that the transmission of an infectious disease, tumor, or other diseases to the recipient was linked to the transplantation of the donor organ into the following categories: proven, probable, possible, unlikely, excluded, or not assessable. The grading system is adapted from the US Disease Transmission Advisory Committee (DTAC) [4, 10]. *Probable* means “the following two conditions are met: Suspected transmission and laboratory evidence of the pathogen or the tumor in a recipient. And it meets at least one of the following conditions: Laboratory evidence of the same pathogen or tumor in other recipients and/or laboratory evidence of the same pathogen or tumor in the donor. If there is pre-transplant laboratory evidence, such evidence must indicate that the same recipient was negative for the pathogen involved before transplant” [4]. *Proven* means that all conditions are met. In the case of only one recipient a clear signature tying the donor and recipient together is necessary (i.e., fluorescent *in situ* hybridization (FISH) or DNA molecular analysis) [4].

In case of malignancy, a donor-transmitted cancer (DTC) was defined as present within the allograft at the time of transplantation and a donor-derived cancer (DDC) as developing within the donor cells following transplantation [5, 15].

## Results

From 1st January 2016 to 31st December 2022, 8,519 organ donors (5,995 organ donors from Germany, 2,524 donors from other European countries) donated 21,060 organs to 20,315 recipients. During this period, a total of 543 serious adverse events (SAEs) and serious adverse reactions (SARs) were recorded by the SAE/SAR team. In 418 (418/543, 77%) cases, the organ donation took place in Germany and in 125 cases (125/543, 23%) the organ donation took place in another European country and at least one recipient in Germany was transplanted.

365 of the reported cases (365/543, 67%) were classified as SAEs and 178 cases (178/543, 33%) as SARs. 336 reports were classified as pathogen/infection (336/543, 62%), 145 as suspected malignancy (145/543, 27%), 40 as other diseases (40/543, 7%), 11 as an immunologic disease (11/543, 2%), and 11 as a genetic disease (11/543, 2%) (Table 1).

**TABLE 1:** Different categories in all reported SAE/SAR cases.

	Total reports	P/P donors	Total recipients from P/P donors	Total recipients with transmission from P/P donors^a^	Total deaths from disease transmission^b^
Bacteria	169	12	43	20 (47%)	0 (0%)
Fungus	114	10	41	11 (27%)	2 (18%)
Virus	48	7	24	13 (54%)	3 (23%)
Parasite	5	1	4	1 (25%)	1 (100%)
Suspected Malignancy	145	16	43	22 (51%)	11 (50%)
Genetic	11	3	10	3 (30%)	0 (0%)
Immunologic	11	1	2	1 (50%)	0 (0%)
Others	40	3	11	4 (36%)	0 (0%)
Total	543	53	178	75 (42%)	17 (23%)

Abbreviation: P/P, proven/probable.

^a^ % = recipients with transmission/recipients from proven/probable (P/P) donors.

^b^ % = death from disease transmission/total recipients with disease transmission.

In the pathogen/infection category, bacteria accounted for 169 cases (169/336, 50%), fungi for 114 cases (114/336, 34%), viruses for 48 cases (48/336, 14%), and parasites for five cases (5/336, 2%) (Table 1). In 68 of the 336 cases, more than one pathogen was found, resulting in a total of 412 pathogens (68/336, 20%).53 donors (53/8,519, 0.62%) transmitted a proven/ probable disease to 75 recipients (75/20,315, 0.37%). 17 of the 75 recipients with proven/probable transmitted disease died as a consequence (17/75; 23%) (Table 1).

The most common bacteria reported were *Staphylococcus* spp. (59 cases, including 16 methicillin-resistant *S. aureus* and 3 methicillin-resistant *S. epidermidis*) followed by *Klebsiella* spp. (25 cases, including 10 multidrug-resistant), *Enterococcus* spp. (21 cases, including 5 vancomycin-resistant *Enterococcus faecium*), *E. coli* (20 cases including 4 multidrug-resistant), *Acinetobacter* spp. (15 cases, including 10 multidrug-resistant), and *Pseudomonas* spp. (15 cases, including 4 multidrug-resistant). There were 7 reports with *Mycobacteria* (4 *Mycobacterium tuberculosis* and 3 *non-tuberculous Mycobacteria*). 67 of the recorded 209 bacteria (67/209, 32%) were multidrug resistant (MDR) pathogens (Table 2).

**TABLE 2:** Summary of bacterial pathogens in all reported SAE/SAR cases.

Bacterial pathogen	All cases	MDR	Donor transmitted P/P	Recipients P/P	Death P/P
*Staphylococcus* spp.	59	19	0	0	0
*Klebsiella* spp.	25	10	3	6	0
*Enterococcus* spp.	21	5	5	10	0
*E. coli*	20	4	2	2	0
*Acinetobacter* spp.	15	10	0	0	0
*Pseudomonas* spp.	15	4	0	0	0
Mycobacteria	7	0	1	1	0
Other Bacteria	47	15	1	1	0
Total	209^a^	67	12	20	0

Abbreviation: spp., species; MDR, multi drug resistant; P/P, proven/probable.

^a^ In 40 cases more than one pathogen.

In 12 cases, bacteria were responsible for a proven/probable transmission of an infection: *Enterococcus faecium* (5 cases including 4 cases with vancomycin-resistant *E. faecium*), *Klebsiella pneumonia* (3 cases), *E. coli* (2 cases), *Streptococcus pneumonia* (1 case), and *Mycobacterium tuberculosis* (1 case). There were 20 recipients with clinical symptoms, but no fatal course (Table 2). In five recipients, the kidney needed to be removed because of a hemorrhage of the infected arterial anastomosis.

In 10 cases, fungi (7 *Candida* spp., 2 *Aspergillus* spp., 1 *Cryptococcus*) were responsible for 10 proven/probable transmission to 11 recipients. Two of them died because of a hemorrhage of a mycotic aneurysm after kidney transplantation, in one additional case the kidney had to be removed. In all three cases *Candida albicans* was detectable at the renal allograft artery. In the case of the transmission of *Aspergillus fumigatus* one recipient developed an intracerebral aspergillosis and another recipient a pulmonary aspergillosis with the need of lobectomy (Table 3).

**TABLE 3:** Summary of fungal pathogens in all reported SAE/SAR cases.

Fungal pathogen	All cases	Donor transmitted P/P	Recipients P/P	Death P/P
*Candida* spp.	91	7	7	2
Aspergillus spp.	15	2	3	0
Mucor	3	0	0	0
Cryptococcus	2	1	1	0
Other	3	0	0	0
Total	114	10	11	2

Abbreviations: spp., species; P/P, proven/probable.

In 7 cases, proven/probable viruses were transmitted to 13 recipients, of which three died. There was one HCV transmission to five recipients, two HEV transmissions to two recipients, one HHV-6 transmission to one recipient (three-year-old child), one HHV-8 transmission to one recipient, and one Borna disease virus 1 (BoDV-1) transmission to three recipients. In the case of the BoDV-1 transmission, two recipients died, and one patient survived with neurological deficits. One recipient died after the transmission of HHV-8 virus from the donor (Table 4).

**TABLE 4:** Summary of viral pathogens in all reported SAE/SAR cases.

Viral pathogen	All cases	Donor transmitted P/P	Recipients P/P	Death P/P
HBV	7	1	1	0
HCV	6	1	5	0
HEV	5	2	2	0
BoDV-1	1	1	3	2
HHV-6	1	1	1	0
HHV-8	1	1	1	1
Other	27	0	0	0
Total	48	7	13	3

Abbreviations: HBV, hepatitis B virus; HCV, hepatitis C virus; HEV, hepatitis E virus; BoDV-1, borna disease virus 1; HHV-6, human herpesvirus 6; HHV-8, human herpesvirus 8; P/P, proven/probable.

In one case, a parasite infection (*Toxoplasma gondii*) was transmitted to one recipient resulting in the death of the patient.

Of the 145 cases categorized as potential malignancy, 104 showed a malignant tumor after the final histopathological examination (104/145, 72%). 16 cases were classified as proven/probable transmission (16/104, 15%) to 22 recipients resulting in 11 deaths (11/22, 50%) (Table 5).

**TABLE 5:** Summary of malignancies in all reported SAE/SAR cases.

Malignancy Type/Location	All cases	Donor derived (DDC)	Donor transmitted P/P (DTC)	Total recipients from P/P donors	Total recipients with P/P Transmission^a^	Death from P/P Transmission^b^
RCC	43	16	2	5	2 (40%)	0 (0%)
Other malignancy	26	1	3	8	4 (50%)	3 (75%)
Adenocarcinoma	11	2	3	8	4 (50%)	3 (75%)
Urothelial carcinoma	9	6	2	8	2 (25%)	0 (0%)
Lung cancer	8	4	2	6	2 (33%)	1 (50%)
Lymphoma	5	0	2	5	4 (80%)	2 (50%)
Melanoma	2	0	2	4	4 (100%)	2 (50%)
Total	104	29	16	44	22 (50%)	11 (50%)

Abbreviations: DDC, donor-derived cancer; DTC, donor-transmitted cancer; RCC, renal cell carcinoma; P/P, proven/probable.

^a^ Recipients with transmission/all recipients from P/P donors.

^b^ Death from P/P transmission/all recipients with P/P transmission.

The mean time until the diagnosis was made in the 22 recipients was 6.3 months (0–36 months). The cases with the proven/probable malignancy transmission included three adenocarcinoma, two lymphoma, two melanoma, two renal cell carcinoma (RCC), two neuroendocrine lung cancer, two urothelial carcinoma, one angiosarcoma, one pleural mesothelioma, and one squamous cell carcinoma.

The most commonly reported malignant tumor was a RCC. RCC accounted for 43 of the 104 cases categorized as malignant (43/104, 41%), 16 of them were donor-derived with a mean time of 7.9 years after transplantation. In two cases a proven/probable transmission of the RCC to two recipients occurred. Also common were adenocarcinoma (11 cases, 3 donor-transmitted), urothelial carcinoma (9 cases, two donor-transmitted), lung cancer (8 cases, two donor-transmitted) and lymphoma (5 cases, two donor-transmitted) (Table 5).

Overall, in the 6 years from 2016 to 2022, 0.19% of the 8,519 donors (16/8,519, 0.19%) transmitted a proven/probable malignancy to 0.11% of all recipients (22/20,315, 0.11%).

There were three proven/probable transmissions of a genetic disease to three recipients: One catecholaminergic polymorphic ventricular tachycardia (CPVT) transmitted to the heart recipient, one hemochromatosis transmitted to the liver recipient, and one factor VII deficiency transmitted to the liver recipient. In the category of other diseases, one donor transmitted a membranous nephropathy to both kidney recipients, one donor transmitted a fibromuscular dysplasia to one recipient, and one donor transmitted a thrombotic microangiopathy to one recipient. Furthermore, one recipient developed an acute graft host versus disease after liver transplantation. None of these patients died.

## Discussion

In the 6 years from 2016 to 2022, donor-derived disease transmission occurred in 0.37% of all recipients (75/20,315; 0.37%). Compared to other risks of transplantation, such as 30-day mortality or delayed organ function, this risk can be considered relatively low. However, in the case of a proven/probable transmission of a disease to the recipient, the mortality is significant (overall 17/75, 23%), and in the case of a malignant tumor (11/22, 50%) [4, 5, 7, 16].

In the DTAC report on cases of potential donor disease transmission events (PDDTE) from 2008 to 2017, 15% of the cases resulted in a proven/probable transmission (335/2,185; 15%) [7]. In our series from 2016 to 2022, we had a rate of 10% proven/probable cases (53/543; 10%).

One explanation for this could be the relatively high proportion of SAE cases (365/543, 67%) in our series. For instance, our cases also included contaminated transportation fluid or antibiotic sensitive blood cultures in the donor. In most cases, there was no infection of the recipients attributable to the reported microorganisms. When the German V&S system was implemented, it was established to document all possible SAEs and SARs in order to learn if there is a clinical impact at all. For this reason, it is possible that our data reflect an overreporting of SAE cases with no relevance to the recipient. Within these years we implemented, together with leading German transplant centers, a “white list” definition of which germs should be reported as SAE. On the other hand, omitting these cases may oversee possibly relevant information with clinical impact to the recipient. The guide to the quality and safety of organs for transplantation [10] and the European Framework for Evaluation of Organ Transplants project (EFRETOS) [11] published lists with detailed examples of various SAEs and SARs.

In the future, a more detailed evaluation of the different pathogens (multiresistant bacteria, fungi) and transmission routes (blood, broncho-alveolar lavage, transportation fluid) may potentially help to better assess the risks for transmission of an infectious disease from the donor to the recipient.

On the other hand, in all publications concerning SAEs and SARs, there is the potential problem of underreporting due to the fact that the reports are dependent on the donor hospitals or transplant centers providing information. Although it is mandatory to report potential SAEs or SARs to the DSO, it is not known exactly how many donor hospitals or transplant centers accomplish this task and adhere to the rule. In Germany (and in the other countries of the EU) there a no legal penalties in case of non-reporting of an SAE/SAR case. Audits of the entire organ procurement process chain including SAE/SAR reporting take place regularly at donor hospitals and transplant centers. However, a systematic monitoring process guaranteeing complete reporting of all SAEs/SARs occurring in the German hospitals and transplant centers is difficult to achieve, considering the almost 1,300 donor hospitals and more than 110 transplant programs in 46 transplant centers. Furthermore, if we compare the incidence of donor-transmitted cancer (DTC) to one or more recipients from our series (22/20,315; 0.11%) with the incidence of other cohorts (0.02%–0.06%) [5, 7, 16, 17] the result is not indicative of serious underreporting. The differences in rates of DTC could reflect a higher age of donors, existing co-morbidities, and a different reporting behavior of an active V&S system compared to a registry. Larger cohorts and longer follow-up times may still be needed [7]. At present, it seems that a combination of an active V&S system and a transplant registry at a national, or even better, at an international level could provide a better assessment of the risk for organ recipients [17].

Implementing an effective and reliable V&S system is essential in order to improve patient safety and transparency in the field of organ donation and transplantation. Different steps are necessary to reach this goal: the awareness of all parties for this topic where SAEs and SARs can occur, fast alert to the responsible institution (in Germany DSO), immediate information of the involved transplantation centers and donor hospitals, initiation of corrective and preventive measurements in the recipients, assessment of the clinical significance of SAEs or SARs, reporting to the medical community and, if appropriate, implementing new guidelines. For instance, recently, the Organ Process Chain Committee (OPCC) of Eurotransplant (ET) sent a letter to the national competent authorities including a list of microorganisms to be reported as SAEs or SARs when found in broncho-alveolar lavage (BAL) or transport fluids based on the German data reported [18]. In addition, patients on the waiting list can be better informed about possible risks of the organ transplantation. For this, all parties of a healthcare system have to be aware of the risk for SAE and SAR in organ donation and transplantation and feel responsible for reporting and sharing these cases. Although there is a legal obligation, there is no perfect “supervising” tool, yet.

At the same time V&S should not be used to punish a hospital when an SAE or a SAR has occurred. This is crucial for the acceptance of this alert system. A no-blame philosophy should lead the communication with all involved institutions and a constructive dialogue based on a partnership should be followed.

Taken together, the goal of an effective V&S system is to create a reliable and rapid alert system to all involved parties of the transplantation community, to assess the risk of transmission of infectious diseases or malignancy from organ donors to the recipients, to improve decision-making in terms of better risk evaluation of the donors, and to improve the safety of donation and transplantation of organs in general.

## Data Availability

The raw data supporting the conclusion of this article will be made available by the authors, without undue reservation.

## Abbreviations

BAL, broncho-alveolar lavage; BoDV-1, borna disease virus 1; CPVT, catecholaminergic polymorphic ventricular tachycardia; DDC, donor-derived cancer; DSO, Deutsche Stiftung Organtransplantation; DTAC, Disease Transmission Advisory Committee; DTC, donor-transmitted cancer; EFRETOS, European Framework for Evaluation of Organ Transplants; ET, Eurotransplant; EU, European Union; FISH, fluorescent *in situ* hybridization; HBV, hepatitis B virus; HCV, hepatitis C virus; HEV, hepatitis E virus; HHV-6 virus, human herpesvirus 6; HHV-8, human herpesvirus 8; MDR, multi drug resistant; OPCC, Organ Process Chain Committee; P/P, proven/probable; PDDTE, potential donor disease transmission events; RCC, renal cell carcinoma; SAE, Serious Adverse Event; SAR, Serious Adverse Reaction; SPP, species; SOP, Standard Operating Procedures; V&S, Vigilance and Surveillance.
